# Child-Like Adults: Dual-Task Effects on Collective vs. Distributive Sentence Interpretations

**DOI:** 10.3389/fpsyg.2021.556120

**Published:** 2021-06-10

**Authors:** Anna M. B. de Koster, Petra Hendriks, Jennifer K. Spenader

**Affiliations:** ^1^Center for Language and Cognition Groningen (CLCG), Faculty of Arts, University of Groningen, Groningen, Netherlands; ^2^Department of Artificial Intelligence, Faculty of Science and Engineering, University of Groningen, Groningen, Netherlands

**Keywords:** conversational implicature, distributivity, dual task, language development, pragmatics, quantification, semantics, working memory

## Abstract

In this work, we consider a recent proposal that claims that the preferred interpretation of sentences containing definite plural expressions, such as “The boys are building a snowman,” is not determined by semantic composition but is pragmatically derived via an implicature. Plural expressions can express that each member of a group acts individually (distributive interpretation) or that the group acts together (collective interpretation). While adults prefer collective interpretations for sentences that are not explicitly marked for distributivity by the distributive marker *each*, children do not show this preference. One explanation is that the adult collective preference for definite plurals arises due to a conversational implicature. If implicature calculation requires memory resources, children may fail to calculate the implicature due to memory limitations. This study investigated whether loading Dutch-speaking adults' working memory, using a dual task, would elicit more child-like distributive interpretations, as would be predicted by the implicature account. We found that loading WM in adults did lead to response patterns more similar to children. We discuss whether our results offer a plausible explanation for children's development of an understanding of distributivity and how our results relate to recent debates on the role of cognitive resources in implicature calculation.

## Introduction

An essential feature of language is the ability to refer to groups of individuals. We can talk about these individuals performing actions individually, or together as a group. Consider sentence (1), which contains the plural definite description *the boys*:

(1) The boys are building a snowman.

**Figure 1:**
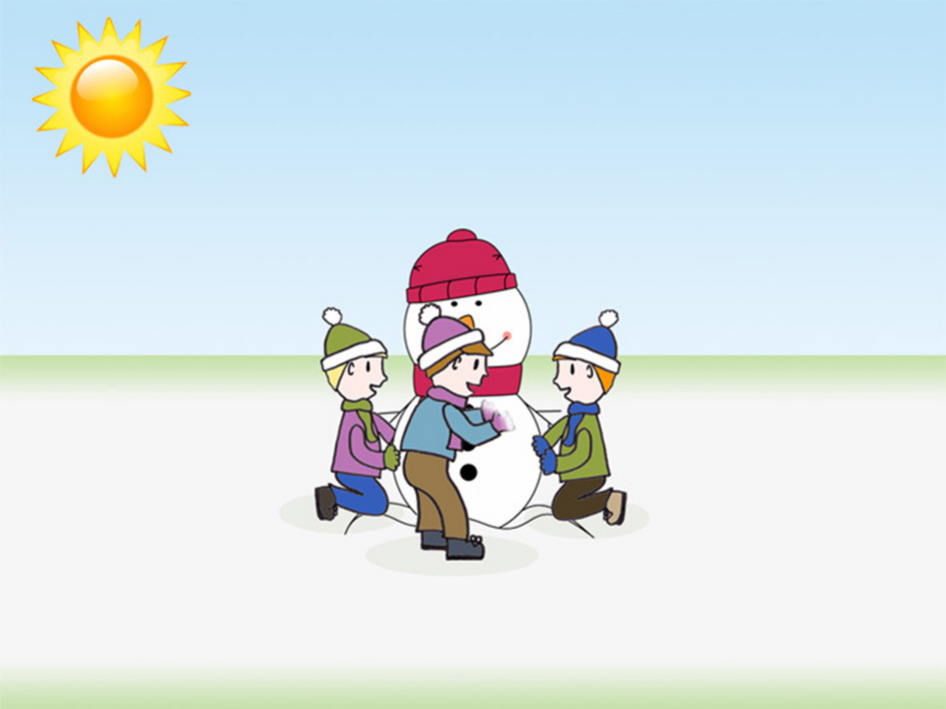
Collective interpretation.

**Figure 2:**
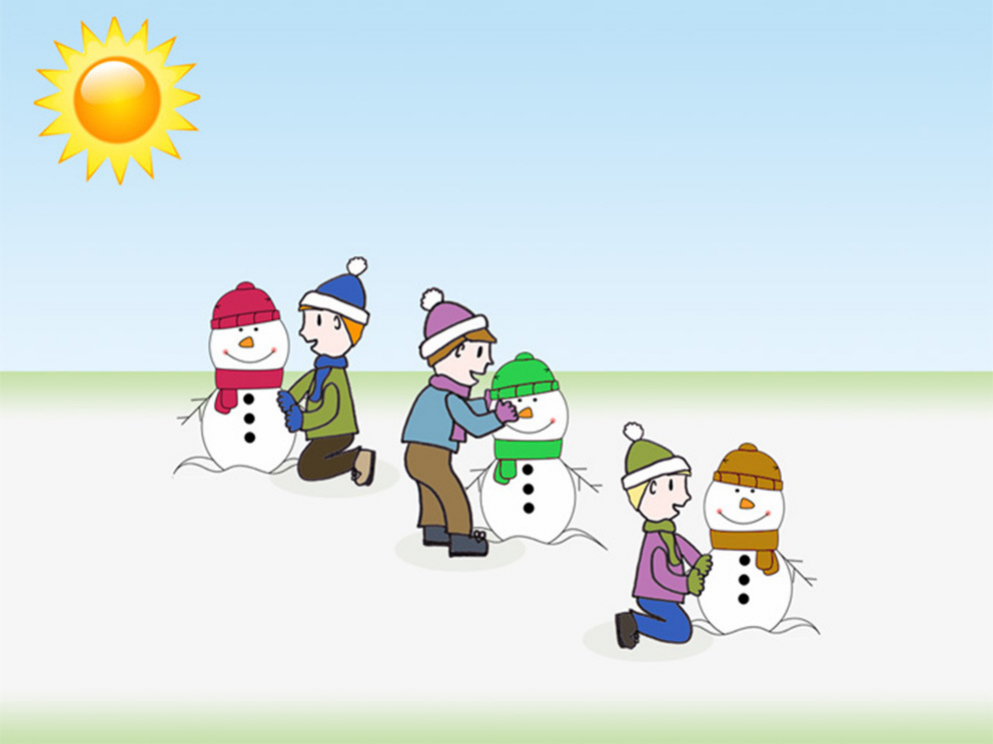
Distributive interpretation.

Sentences with plural definite subjects like (1) are compatible with more than one interpretation according to semantic theories (e.g., Landman, 2000; Champollion, 2017). Are the boys in sentence (1) building one snowman together (the collective interpretation, see Figure 1)? Or are they building individual snowmen (the distributive interpretation, see Figure 2)? A collective interpretation simply requires that the predicate applies to the set denoted by the plural expression. For example, in a situation with three boys, Al, Ben, and Chris, the collective interpretation of the sentence “Every boy is building a snowman” means that several of the boys are building one snowman together. The distributive interpretation, on the other hand, requires that the predicate applies to each member of the set denoted by the plural expression individually. This would necessarily entail that Al is building his own snowman, Ben is building his own snowman, and Chris is building his own snowman.

Experimental studies have shown that adults disprefer distributive interpretations, unless an overt distributive marker like *each* is present (Gil, 1982; Brooks and Braine, 1996; Frazier et al., 1999; Kaup et al., 2002), as in (2). When *each* is present, adults prefer distributive interpretations almost exclusively.

(2) Each boy is building a snowman.

The preference for a collective or distributive interpretation is affected by multiple other factors as well, such as whether or not the action tends to be done with others, e.g., carrying a piano, or tends to be done individually, e.g., drinking an espresso [see also Geurts (2010); de Koster et al. (2020); Kursat and Degen (2020) for more discussion]. However, the presence or absence of distributive marking seems to be the most influential feature for lexically ambiguous predicates [see (Dotlačil and Brasoveanu, 2021) for recent results].

Studies in various languages have shown that children have different preferences than adults when interpreting plural NPs like (1). Whereas, adults prefer collective interpretations, children seem to initially prefer distributive interpretations across the board, even when no distributive marker is present (Miyamoto and Crain, 1991; Avrutin and Thornton, 1994; Brooks and Braine, 1996; Syrett and Musolino, 2013). In fact, we can identify two milestones in children's development of adult-like preferences. First, around age-seven children begin to consistently reject distributive markers like *each* with collective situations (Pagliarini et al., 2012; de Koster et al., 2017, 2018), suggesting that it is not until that age that children fully grasp the distributive import of distributive markers. Second, around the age of nine children begin to reject distributive interpretations if there is no distributive marking, like adults. However, they initially reject these cases at a much lower rate than adults. The rate of rejection increases steadily with age, but existing studies found that at age 11 [de Koster et al. (2018) for Dutch] and even at age 14 [Pagliarini et al. (2012) for Italian], the rates of rejection were still lower than those of adults in the same study.

In this paper, we experimentally investigate a proposal that would simultaneously explain adults' rejection of distributive interpretations with distributively unmarked sentences and, perhaps, offer an account for children's development of adult-like preferences: The implicature account of distributivity preferences developed by Dotlačil (2010). This account proposes that the preference to interpret distributively unmarked sentences as collective results from a conversational implicature. To investigate this proposal, we focus on whether or not adult preferences require working memory resources. We focus on the potential role of working memory resources in adults' distributivity preferences for two reasons. First, many studies of more established conversational implicatures have found evidence that calculating an implicature requires working memory resources. Second, in a study of children's distributivity preference development, de Koster et al. (2018) found a positive correlation between working memory capacity and adult-like preferences, suggesting that working memory plays a role in children's distributivity preferences. We thus investigate the role of working memory in distributivity preferences by limiting adults' working memory with a dual-task design. If limited working memory leads to responses more similar to children, we will have found evidence supporting an implicature account of distributivity and a role for working memory in children's non-adult preferences.

## Implicatures and Processing

Conversational implicatures are language-based inferences about a speaker's intended meaning that listeners make by considering alternative forms the speaker could have used (Grice, 1975). The most researched implicature is triggered by the scalar term “some.” Consider (3):

(3) Teacher: “Some of my students passed the exam.”

Semantically, (3) is consistent with several students passing the exam, but also with all the students passing the exam, because *some* literally means “at least one.” However, most listeners understand (3) to mean that not all students passed, because they will not simply interpret what the speaker literally says, but they will compare it with alternatives that the speaker could have said but did not. Because listeners expect speakers to use the most informative expression consistent with the situation, the choice by the speaker to use a weaker expression will suggest to the listener on the comparison that the stronger expression did not hold. Because *all* semantically entails *some, all* is informationally stronger than *some*, so an utterance with the weaker form *some* then implicates that the speaker believes that the stronger version with *all* does not hold (Horn, 1973). Because this explanation relies on the recognition of a scale of informativity (e.g., <some, all>, <might, must>, <or, and>), these are also often called scalar implicatures.

Conversational implicatures are pervasive in language: Strengthened meanings are associated with specific scalar lexical items like *some* or *most* but also can be calculated on the fly. E.g., in the following short exchange, A: *Did you read War and Peace?* B: *I read the first chapter*, B's statement implicates that B did not read the rest of the book.

Dotlačil's (2010) proposal that sentences with definite plurals are interpreted collectively due to an implicature builds on the accepted view that distributively unmarked sentences, such as sentences with definite plural subjects, can semantically express both a collective and a distributive interpretation (Frazier et al., 1999). In contrast, an utterance with the distributive marker *each*, such as sentence (2), signals the more specific distributive interpretation and is thus informationally stronger than a distributively unmarked utterance like (1), which is ambiguous. A distributively unmarked sentence such as (1) is not specified for collective or distributive meaning, but will be interpreted as collective, because a hearer will reason that if the speaker had intended a distributive interpretation, the speaker would have used the informationally stronger form with the distributive marker *each*. Through the reasoning process underlying implicatures, unmarked sentences are biased to be interpreted collectively.

Note that this account proposes that the collective and the distributive interpretations have different sources: Distributive interpretations arise due to distributive marking, such as *each*, while collective interpretations arise because of the absence of a distributive marker due to an implicature. Because the implicature calculation requires comparing alternatives and, perhaps, others steps not necessary for a literal interpretation, it may involve greater cognitive effort. For this reason, the implicature account offers an explanation of children's non-adult interpretation preferences in terms of processing difficulties.

There are two aspects of the implicature proposal that benefit from a closer examination. First, can distributively unmarked sentences with plural definite descriptions and distributively marked sentences with *each* be analyzed analogously to more traditional implicatures such as *some-not all*? Second, what evidence is there that implicature calculation requires additional cognitive resources, in particular working memory resources?

To see if parallels exist between the well-studied implicature with *some* and *all* and the proposed implicature with plural definites and *each*, let us consider the sentence meanings involved in the implicature calculation in both cases. The set of situations where sentences with *all*, expressing an exhaustive meaning, are true is a proper subset of the set of situations where sentences with *some* under its literal interpretation, “some and possibly all,” are true. Thus, literal *some* can be considered to be less informative than *all*: It allows the “some but not all” meaning as well as the exhaustive “all” meaning. More informative sentences with *all* will block the exhaustive meaning with ambiguous *some* so that it is preferably interpreted only as “some but not all,” resulting in a pragmatically strengthened non-exhaustive meaning for sentences with *some*.

In a similar fashion, plural definite descriptions and distributively quantified DPs can be analyzed. Plural definite descriptions have a so-called “maximality requirement”: the maximal set of the plurality modified by the definite description needs to participate in the predicated action, e.g., in (1) all members of the set of boys must build a snowman. *Each* as a universal quantifier also requires that all members of the restrictor set modified by the quantifier participate exhaustively in the predicated action, e.g., in (2), all members of the set of boys must also participate in snowman building. As a distributive quantifier, *each* imposes an additional distributive requirement, and the result is that the set of situations where sentences with the distributive quantifier *each* (expressing a distributive meaning) are true is a proper subset of the set of situations where sentences with the definite article *the* are true, and the proposition in (2) entails the less specific (1). As such, sentences with *the* can be considered to be less informative than sentences with *each*: They allow a collective as well as a distributive interpretation (e.g., Maldonado et al., 2019). The resulting scale (<plural *the, each*>) also fulfills an additional requirement, emphasized by van Tiel et al. (2019) and originating from Horn (1989), that members of the scale must have the same polarity in that both are positive. According to the proposed distributivity implicature, the more informative sentences with *each* will block the distributive meaning for the less informative sentences with *the*, resulting in a pragmatically strengthened collective meaning for sentences with *the*.

Essential to this analysis is that an implicature with plural definites would only be evoked if a speaker was aware of the unambiguous, distributive import of distributive markers and treat <plural *the, each*> as forming a scale. We expect adults to know what lexical items signal distributivity, but children have to learn this. Therefore, the proposal predicts that only children that understand the distributive meaning and the way this meaning can be marked have the prerequisite knowledge for the implicature. Children are generally considered to understand the meaning of distributive markers if they consistently reject them with collective situations, which has been found experimentally to be around age seven (Pagliarini et al., 2012; de Koster et al., 2017, 2018). Only after this age will they be able to infer that the absence of distributive marking conversationally implicates collectivity. This prediction has been previously experimentally investigated by Pagliarini et al. (2012) in Italian, de Koster et al. (2017) in Dutch, and Padilla-Reyes (2018) in Spanish. All three studies found a correlation between children's rejection of collective situations with distributive marking and children's rejection of distributive situations when distributive marking is not present, consistent with the predictions of the implicature account.

Why would implicature calculation be effortful? And what experimental evidence supports this claim? Implicature accounts differ as to whether they see it as a primarily pragmatic (e.g., Gricean) process, where implicature is the product of the listener's expectation that speakers will be as informative as possible, or as a semantic process (Chierchia, 2004, 2006), where strengthened meaning is argued to originate from an unpronounced operator, O, that signals that stronger alternatives do not apply [i.e., “[O]nly” the literal meaning is intended]. Despite these different assumptions, both pragmatic and semantic accounts of implicature identify at least two steps in implicature calculation that could be potentially cognitively demanding: The decision whether or not to calculate an implicature and the derivation and comparison of alternatives, both considered to be pragmatically driven processes (see, Geurts, 2010; e.g., Chemla and Singh, 2014).

Multiple studies have, in fact, found evidence that verifications of sentences with *some-not all* implicatures take more time than the lower-bounded, literal interpretation of *some*. This has been shown with timed sentence verification (Bott and Noveck, 2004), self-paced reading (Breheny et al., 2006; Chemla and Bott, 2013), eye-tracking (e.g., Huang and Snedeker, 2009), mouse tracking (Tomlinson et al., 2011), and looking at response times, with speed accuracy trade-off (SAT) methods (e.g., Bott et al., 2012). Most relevant for our research, however, are the experiments that focused on determining if there is a memory cost involved in the calculation of scalar implicature, using dual-task designs. These experiments ask the participants to judge sentences while their working memory is loaded (De Neys and Schaeken, 2007; Dieussaert et al., 2011; Marty and Chemla, 2013; Marty et al., 2013; van Tiel et al., 2019; Ryzhova and Demberg, 2020). Most of these studies had participants memorize dot patterns on a 3 × 3 matrix, which they then had to recreate after judging sentences that could invoke an implicature. Most studies compared linguistic performance on low-working memory load patterns, with a systematic three-dot pattern (all horizontal or all vertical), to performance with high-load patterns, which had four dots. De Neys and Schaeken (2007) found that participants under a high-working memory load made significantly fewer *some-not all* implicatures (around 10% less) compared to a low-working memory load. Dieussaert et al. (2011) used an identical design but investigated the role of individual working memory capacity by also measuring participants' working memory spans in a separate task. They found fewer implicature calculations under a high load but only for participants with low-working memory capacity. Marty and Chemla (2013) also used a similar design and found that loading working memory decreased the rate of implicatures but had no effect on semantically equivalent *only some* sentences where the negation of the alternative is made explicit, a result that is unexpected under a semantic account of implicature^1^.

The type of implicature may also influence whether or not working memory resources are involved. Marty et al. (2013) used a dual-task design with a backward letter sequence reproduction task. They found a decrease in implicature interpretations with *some-not all* implicatures under a high memory load but found no effect with number items, which should implicate an exact interpretation (e.g., *three* means *three and no more*)^2^. In a recent study, van Tiel et al. (2019) used a dual-task design to investigate several scalar words, using a between-subjects design to compare participants under no load, a low working memory load, and a high working memory load. They found that the influence of loading working memory varied by implicature type: Some implicatures, such as *some-not all*, showed a lower rate of calculation already in the low-load condition compared to the no-load condition, whereas others only showed an effect between the low-load and the high-load conditions. Ryzhova and Demberg (2020) also carried out a dual-task study, using dot-tracking as the secondary task. Participants made fewer particularized conversational implicatures under a high memory load compared to a low-memory load. In summary, the existing dual-task studies have all found that loading working memory with a dual task lowers the rate of implicature calculation.

In contrast to the dual-task studies, which focused on working memory resources, a number of other studies, which are primarily eye-tracking studies, have failed to find evidence that implicature calculation requires additional cognitive resources. In a visual-world study, Grodner et al. (2010) failed to find that *some-not all* implicature calculations required greater processing times when they modified the materials from (Huang and Snedeker, 2011). Politzer-Ahles and Fiorentino (2013), Hartshorne and Snedeker (2014), in an eye-tracking reading experiment, and Politzer-Ahles and Husband (2018), all also failed to find evidence of additional processing costs for implicatures. For implicatures based on the <*not all, none*> scale, Cremers and Chemla (2014) (Exp. 1) and Romoli and Schwarz (2015) both found that participants were faster at implicature calculation than the literal interpretation. In a visual world eye-tracking study, Degen and Tanenhaus (2016) found no difference between pragmatic and literal interpretations of *some*. Kursat and Degen (2020), investigating reaction times with *some-not all* implicatures found evidence for two populations: pragmatic responders who tended to always calculate implicatures and who were faster at these interpretations than at lower-bounded interpretations, and literal responders. Like the results from Dieussaert et al. (2011), this shows that there may be individual differences in implicature calculation tendencies.

Another issue in experimental implicature research is that many studies focus on participants' end-state judgments without further information about the interpretation process. Because end-state judgments are a culmination of multiple interpretation processes, both semantic and pragmatic,^3^ it can be hard to determine what influences the participants' final judgment.

If implicatures do require additional resources, exactly what resources and at what step in their interpretation these become relevant is still a topic of investigation. Dotlačil's (2010) implicature account of distributivity preferences claims to both explain adults' preferences and offer an account for children's non-adult preferences by attributing them to children's difficulties in calculating implicatures. Dotlačil does not make specific claims about what aspect of distributivity interpretation preferences might require processing resources. But because many experimental studies did find a role for working memory in implicature calculation, and because de Koster et al. (2018) found that children's tendency to reject distributive situations without distributive marking was found to be positively correlated with these children's working memory capacity, we decided to investigate the role of working memory in distributivity preference in adults.

If we find that limiting working memory capacity in adults decreases their rates of acceptance of distributive readings with distributively unmarked sentences, then children's tendency to allow distributive readings with distributively unmarked sentences might be explained as a consequence of their lower working memory capacity. This result would then be consistent with the predictions of Dotlačil's implicature account of distributive preferences. If we fail to find an effect of limiting working memory capacity, then this would not rule out an implicature calculation if there is no processing cost for implicature (as some research has found). However, this would make the explanation less attractive in that it would fail to offer a processing explanation for children's non-adult interpretation preferences.

We designed our experiment along the lines of previous dual-task experiments, investigating the influence of working memory on implicature calculation. To our knowledge, we are the first to study distributivity interpretations while limiting working memory capacity. Our study thus provides novel empirical evidence, illuminating the role of memory in the interpretation of plural definites.

For practical reasons, we carried out our experiment in Dutch. English has two distributive quantifiers: *each* and *every*. *Each* and *every* are both universal quantifiers that are compatible with distributivity. Whereas *every* only requires partial distributivity, *each* requires full distributivity (Tunstall, 1998), in that a distributive sentence like (2) must entail that for each individual member of the set of boys, it must hold that he was building a snowman. Dutch also has two distributive quantifiers, *elke* and *iedere*. However, experimentally, these quantifiers have been shown to have the exact same interpretation with respect to distributivity preferences (van der Ziel, 2012; Spenader and Bosnic, 2018), so we will simply use *elke*. Research also suggests that both are more similar to *every* than *each*, being partially distributive (Tunstall, 1998) and thus compatible with collective situations in some cases (Rouweler and Hollebrandse, 2015; de Koster et al., 2017). For this reason, we expect that we will see greater acceptance rates of *elke* with collective situations than has been found for *each*, which is fully distributive.

## Method

### Participants

Fifty-eight students from the University of Groningen were paid to participate. They were divided into two groups: a WM Load group (42 participants; 13 men; mean age, 21.9; age range, 18–27) and, to establish a baseline for performance, a No WM load group (16 participants: six men; mean age, 23.9; age range, 20–28). All participants were native speakers of Dutch. This study was carried out in accordance with the recommendations of the Research Ethics Committee (CETO) of the Faculty of Arts of the University of Groningen, and they approved the protocol. We also obtained written informed consent from all the participants prior to testing.

### Design

The WM Load group carried out a dual-task experiment, consisting of two tasks, a linguistic task and a memory load task: while participants interpreted a sentence (the linguistic task), we manipulated their WM load by asking them to memorize a sequence of digits (the digit-span task). For this group, the experiment had a 2 × 2 × 2 design with the factors picture [collective (Figure 1) vs. distributive (Figure 2)], sentence (*de* “the” vs. *elke* “each”) and wm load (low vs. high).

The No WM load group received the linguistic task without the digit span task. This group was not tested in a WM Load condition and, therefore, received the experiment in a 2 × 2 design, with the factors picture and sentence but without the factor wm load. The remaining procedure was the same for the two groups.

#### Linguistic Task

The linguistic task was a sentence–picture verification task. Participants saw a picture on the computer screen and had to judge whether it matched a recorded sentence by pressing a key on the keyboard.

#### Digit-Span Task

At the start of each trial, participants had to memorize a sequence of three or six digits (low and high WM load conditions, respectively), presented on screen for 1 s each. Digits were randomly chosen from 1 to 9, and consecutive digits always differed. After each linguistic item, participants had to recall the digits by typing them in the same order as they appeared.

### Materials

The materials consisted of four practice items, 64 test items, 48 implicature control items and 16 task control items (eight true and eight false items), resulting in a total of 132 items. The experiment was divided into two blocks (preceded by four practice trials), with 64 items per block. The low (three digits) and high (six digits) WM load conditions were presented in different blocks. Block order (either low or high WM load in the first block) was a between-subject factor. The Latin-square design of the test items, together with the factor block order, resulted in eight lists. Item order was randomized for each participant, and the participants were randomly assigned to a list.

#### Test Items

The 64-test items tested the factors picture [collective (Figure 1) vs. distributive (Figure 2)] and sentence [*de* “the” (4) vs. *elke* “each” (5)].

(4) De jongens bouwen een sneeuwpop.
  *The boys are building a snowman*.
(5) Elke jongen bouwt een sneeuwpop.
  *Each boy is building a snowman*.

Eight different transitive verbs were used: build, wash, push, pull, carry, lift, hold or paint (in Dutch: *bouwen, wassen, trekken, duwen, dragen, tillen, vasthouden, verven*), and the grammatical subjects and objects of these verbs varied across the items. The design resulted in four conditions: The-Collective, The-Distributive, Each-Collective, and Each-Distributive. The items of condition The-Distributive test proposed implicature. Each participant saw 16 items in each of the four conditions, resulting in 64 test items (32 items per block).

#### Implicature Control Items

In addition to the test items, participants also received 48 implicature control items to mask the goal of the experiment and to be able to compare the results of the test items to the results of the well-investigated *some-not all* implicature in a dual-task setting.

(6) Sommige jongens vissen.
  *Some boys are fishing*.
(7) Enkele meisjes dansen.
  *Some girls are dancing*.

The implicature control items consisted of two sentence types with the scalar expressions *sommige* “some_1_” (6) or *enkele* “some_2_” (7) (24 items per sentence type). In contrast to *enkele*, which merely expresses existential quantification (“there are some…”), *sommige* additionally indicates that the individuals introduced by the quantifier have something in common that distinguishes them from other individuals (de Hoop and Kas, 1989; Banga et al., 2009). For each item, a different intransitive verb was used (e.g., fishing, dancing, singing, and sleeping).

Both sentence types were combined with four picture types where either zero, one, two or three (i.e., all) of the three actors are performing the action denoted by the sentence. This resulted in eight implicature control combinations. The participants received six items per combination (three items per block). The implicature control items were not constructed via a Latin-square design: All the participants received the same sentence-picture combinations as implicature control items. The examples of the implicature control items are presented in the Supplementary Material.

The implicature control items with a picture with three actors serve as a control to test whether the participants generate a *some-not all* implicature and whether or not this implicature generation is affected by the dual-task setting.

#### Task Control Items

The participants also received 16 task control items. These control items were straightforwardly true or false items and were used to check the participants' attention as well as general task effects such as a possible “yes”-bias. Examples of a true (8) and a false (9) task control item are presented below. The corresponding pictures for items (8) and (9) can be found in the Supplementary Material. The experiment contained eight true task control items and eight false task control items. If the participants answered more than 25% of the task control items incorrectly, they were excluded from the analysis.

(8) De jongen drinkt een pakje melk.
  *The boy is drinking a carton of milk*.
(9) Het meisje drinkt een glas limonade.
  *The girl is drinking a glass of lemonade*.

### Procedure

The participants performed the experiment in a quiet room at the University of Groningen. Participants were shown the pictures on the computer screen while the sentences were played via a speaker. The experimenter was present during the entire experiment.

The experiment started with instructions and four practice trials. For each trial, the participants first saw a digit sequence on screen, followed by a picture and a recorded sentence. The recorded sentence was played only once. They then had to judge sentence acceptability by pressing a green (accept) or red (reject) key. Finally, they had to type in the memorized digits. Participants had 10 s to judge the sentences, with a visual warning message after 7 s. Next, they had 5 s to recall the digits in the low WM load condition and 10 s in the high WM load condition. Pilot testing had shown that this provided the participants with sufficient time.

Each trial ended with feedback to participants on how many digits were recalled correctly. A waiting penalty ensured that participants focused on the WM task and prevented rushing: One incorrect digit resulted in a 1-s waiting penalty, two incorrect digits in a 2-s waiting penalty, etcetera. Self-paced breaks were provided after every 16 items, and the participants had a forced break of at least 2 min in between the two blocks.

The procedure for the two participant groups was similar, including breaks, with the exception that the No WM load group only received the linguistic task. Per trial, the following measures were collected: Accuracy of reproducing the digits in the digit-span task, and yes/no responses and response times for the test items, implicature control items, and task control items in the linguistic task.

### Predictions

The linguistic task tests four conditions: The-Distributive, Each-Collective, The-Collective, and Each-Distributive.

Condition The-Distributive tests whether working memory limitations play a role in children's non-adult acceptance of distributive readings with unmarked sentences. This condition, therefore, also tests the implicature account of distributivity preferences. A “yes” response (acceptance) in this condition would be consistent with a literal interpretation of the distributively unmarked sentence, and a “no” response (rejection) would be consistent with the derivation of an implicature. If there is an effect, we also expect the participants in the No WM load condition and the participants under a low WM load to show a higher rate of rejection than the participants under a high WM load. This result would then be parallel to previous findings for the “some-not all” implicature (De Neys and Schaeken, 2007; Dieussaert et al., 2011; Marty and Chemla, 2013; Marty et al., 2013). This result would also be consistent with the child language data, showing that rejection of the The-Distributive condition in children correlates with working memory capacity (de Koster et al., 2018). Importantly, this is the only condition we expect to be affected by WM load. In addition, we also expect to see higher response times for pragmatic responses compared to literal responses, following the findings of Bott and Noveck (2004).

We do not expect condition Each-Collective to be affected by WM load, and we expect the items of this condition to be rejected due to the distributive character of *each*. Experimental results show that from around age 7;0, children are adult-like in their responses to distributively marked sentences with collective situations. Earlier non-adult acceptance is attributed to children being unaware of the distributive nature of *each*. However, it should be noted that the Dutch *elke* (tested in the current study), contrary to its English counterpart *each*, is only partially distributive and has been found to be more acceptable in collective situations (Rouweler and Hollebrandse, 2015; de Koster et al., 2017). Acceptances of items in this condition are, therefore, not unexpected but are predicted to be independent of WM load.

Condition The-Collective is predicted to be unaffected by WM load too. Sentences with *the* are semantically ambiguous between a collective and a distributive interpretation, so the collective interpretation is a semantically appropriate interpretation for sentences with plural definite subjects. This prediction is supported by child language data, showing that children fully accept *the* sentences with a collective interpretation from age 4 and onward (Italian: Pagliarini et al., 2012; Dutch: de Koster et al., 2017; Spanish: Padilla-Reyes, 2018). The implicature account of distributivity preferences does not predict that a WM load would have an effect on this condition either.

Finally, condition Each-Distributive is also predicted to be unaffected by a WM load. The items in this condition are predicted to be fully accepted since the distributive quantifier *each* is fully compatible with the distributive interpretation. Child language data show children from age 4 until age 14 fully accept distributive interpretations for sentences with *each* (Pagliarini et al., 2012; de Koster et al., 2017; Padilla-Reyes, 2018), which is expected because *each* is semantically distributive.

The implicature control items, testing the “some-not all” implicature, can be used to make a comparison with the results of previous studies testing this implicature (De Neys and Schaeken, 2007; Dieussaert et al., 2011; Marty and Chemla, 2013; Marty et al., 2013). The items of our implicature control “Some-All” condition consist of sentences with *sommige* “some” in combination with a picture in which all the actors are performing the action denoted by the predicate. A “yes” response in this condition indicates a literal interpretation, in which the “*at least one and possible all*” meaning of “*some*” is accepted. A “no” response, on the other hand, indicates a pragmatic interpretation in which *some* is interpreted as “*some but not all*.” We expect to see fewer pragmatic “no” responses under a high WM load, similar to the findings of previous studies (De Neys and Schaeken, 2007; Dieussaert et al., 2011; Marty and Chemla, 2013; Marty et al., 2013). Following the findings of Bott and Noveck (2004) and others, we also expect to observe longer response times for these responses because calculating an implicature comes at a cost.

## Results

Two participants of the WM Load group were excluded from the analysis: One participant did not complete the experiment due to technical problems, and one participant gave incorrect answers to more than 25% of the task control items. All remaining participants were included in the analysis.

### Digit-Span Task

The WM Load group participants remembered 94% of the digits correctly in the low WM load condition (three digits) and 75% of the digits in the high WM load condition (six digits). This drop in performance is significant [paired-t_(39)_ = 13.873; *p* < 0.001], indicating that the high WM load condition was, indeed, more difficult. Furthermore, the linguistic condition had no effect on the percentage of correctly recalled digits. This shows that the participants focused on digit recall performance throughout the experiment, irrespective of linguistic condition.

### Linguistic Task

#### Responses to Test Items

**Figure 3:**
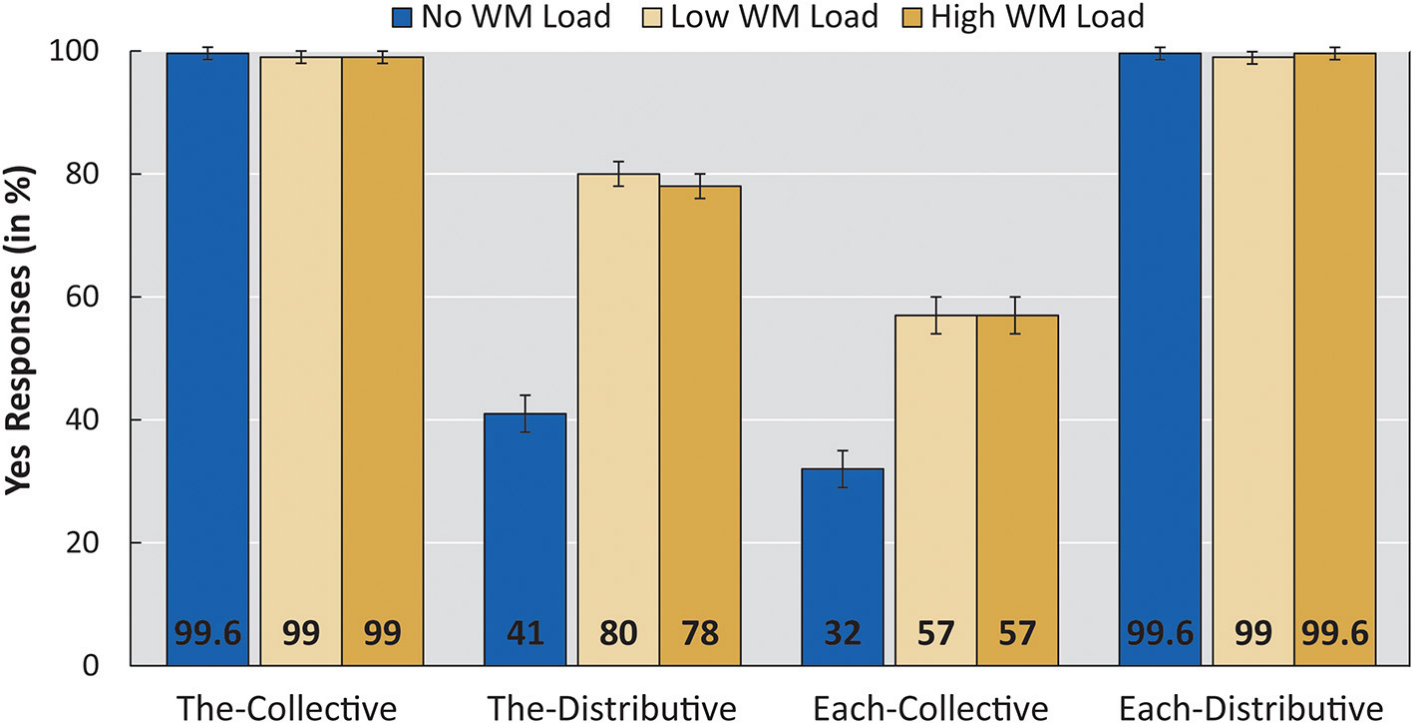
Mean acceptance rates per linguistic condition per WM load (No load, Low, High). Error bars show standard error. The WM Load group was tested with Low and High WM load; the No WM load group was tested on the linguistic task only. Sentences contained either *de* “the” or *elke* “each” and pictures showed either a collective action or a distributive action.

Figure 3 shows the mean acceptance rates for all four linguistic conditions per WM load. The collected data were analyzed, using generalized mixed effect logistic modeling [function glmer(): lme4 package in R, version 3.6.3].

The models were constructed via an iterative forward fitting procedure with model comparisons (cf. Baayen et al., 2008; Wieling et al., 2011; Ko et al., 2014) based on the evaluation of Akaike's information criterion (AIC) (cf. Burnham and Anderson, 2002; Akaike, 2011; Ko et al., 2014; Wieling et al., 2014). An AIC decrease of more than two indicates that the goodness of a fit of the model improves significantly (Akaike, 2011). The AIC values were obtained via model comparisons, using ANOVA testing [function anova() in R]. We determined whether the following fixed-effect factors improved the goodness of fit of the model: sentence (each, the), picture (collective, distributive), block (first, second), wm load (no load, low, high), and verb. The dependent variable was the response (0 for rejection, 1 for acceptance). The final model (Table 1) included the fixed factors sentence, picture, wm load, and block. The factor verb did not significantly improve the model fit and was left out. The maximal random-effects structure licensed by the data included a random intercept for participants, items and by-participant random slopes for sentence, picture, and block.

**Table 1:** Overview of the final model for the responses to the test items, with reference levels: Sentence: “De” “The,” Picture: Distributive, WM Load: No Load, and Block: 1.

**Predictors**	**Estimate (β)**	**SE**	***z***	***p***
Intercept	0.3307	0.844	0.695	<0.001
Sentence “Elke” “Each”	13.316	2.528	5.267	<0.001
Picture collective	9.470	2.193	4.319	<0.001
WM load low	2.809	1.049	2.679	<0.01
WM load high	2.730	1.048	2.605	<0.01
Block 2	−0.419	0.356	−1.180	0.238
Sentence “Elke” × Picture Collective	−24.392	3.584	−6.807	<0.001

A main effect of sentence and picture was found, as well as an interaction between the two. Crucial to our findings is the significant difference between the No WM load group and each of the low and high WM load conditions of the WM Load group (low: β = 2.809; z = 2.679; *p* = 0.007, high: β = 2.730; z = 2.605; *p* = 0.009). In line with our predictions, participants accepted significantly more items of condition The-Distributive in the WM load group compared with the No WM load group.

A releveled model revealed no significant difference between the low and high WM load conditions within the WM load group (β = −0.079; z = −0.235; *p* = 0.814). The low and high WM load conditions had acceptance rates of condition The-Distributive to the same degree.

To check for an influence of the factor block-order (low or high WM load in the first block), we performed a separate analysis on the data of the WM load group, with a similar model. In this model, block-order did not improve the model fit. This shows that participants' acceptance of The-Distributive items was not influenced by whether they received the low or high WM load condition first. Note that it is not possible to add the factor block-order to the final model presented in Table 1 (analyzing the WM load and the No WM load group together) for reasons of collinearity.

#### Responses to Implicature Control Items

**Figure 4:**
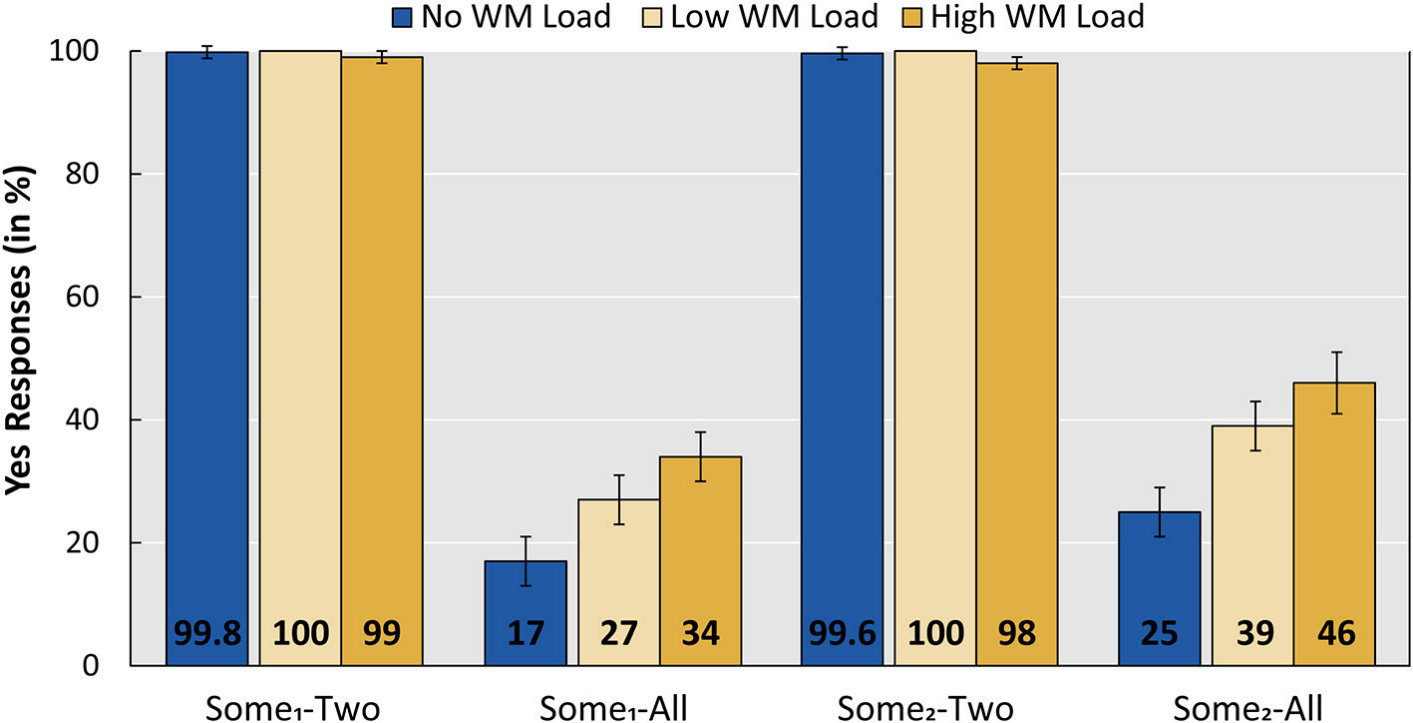
Mean acceptance rates per implicature control condition per WM load (No load, Low, High). Error bars show standard error. The WM Load group was tested with Low and High WM load; the No WM load group was tested on the linguistic task only. Sentences contained either *sommige* “some1” or *enkele* “some2,” and pictures showed either two out of three actors or all three actors performing the action denoted by the sentence.

We also analyzed the responses of the implicature control items with *sommige* “some_1_” and *enkele* “some_2_” in combination with pictures where three (i.e., all) actors are performing the action denoted by the sentence, to check how our WM manipulation affected the “some-not all” implicature. These items are compared to implicature control items with pictures where two actors are performing the action denoted by the sentence, since these latter items do not give rise to an implicature and should be considered true. Figure 4 presents the results for these items. The remaining implicature control combinations (with pictures with zero actors or one actor) were omitted from the figure and further analysis, since they are expected to be judged as false, because sentences with Dutch *sommige* “some_1_” and *enkele* “some_2_” require reference to at least two individuals (Broekhuis and den Dikken, 2012, p. 895).

Models were again constructed via an iterative forward fitting procedure. Using model comparisons (cf. Baayen et al., 2008) based on the AIC values, we determined which fixed-effect factors would improve the model fit. The final model (Table 2) included the fixed factors sentence, picture, and wm load. The dependent variable was the response (0 for rejection, 1 for acceptance). The maximal random-effects structure licensed by the data included a random intercept for participants and by-participant random slopes for sentence and picture.

**Table 2:** Overview of the final model for the responses to the implicature control items, with reference levels: Sentence: “Sommige” “Some_1_,” Picture: All, WM Load: high.

**Predictors**	**Estimate (β)**	**SE**	***z***	***p***
Intercept	−1.849	0.755	−2.447	<0.05
Sentence “Enkele” “Some_2_”	1.448	0.456	3.177	<0.01
Picture two	9.163	1.636	5.601	<0.001
WM load no load	−2.188	1.089	−2.009	<0.05
WM load low	−0.653	0.311	−2.099	<0.05

A main effect of sentence and picture was found, but an interaction between the two did not improve the model fit. Crucial, however, is the significant difference between the high WM load condition (six digits) and both the low WM load condition (three digits) and the No WM load group (low: β = −0.653; z = −2.099; *p* = 0.0358, no load: β = −2.188; z = −2.009; *p* = 0.0445). In line with the predictions regarding the “some-not all” implicature, participants accepted significantly more items of condition Some_1_-All (accepting the literal interpretation of “some”) under a high WM load. A releveled model with “some_2_” *enkele* as the reference level revealed that a similar pattern holds for condition Some_2_-All.

An additional releveled model (with the low WM load as a reference level) revealed no significant difference between the low WM load condition and the No WM load group (β = 1.535; z = 1.421; *p* = 0.155). This means that the participants' calculation of “some-not all” implicatures was only affected by the high WM load condition of six digits.

#### Responses to Task Control Items

The task control items (straightforwardly true or false items) were included to check for participants' attention to the linguistic task. Overall, participants answered 95% of all task control items correctly, which shows that they paid sufficient attention to the linguistic task.

To investigate whether the difference we found in acceptance rates between the WM load group and the No WM load group for the test items could be attributed to a general tendency to more readily accept items when WM is loaded, we also analyzed participants' performance on the false task control items (that required a “no” response).

The WM load group participants answered 89% of the false control items correctly in the low WM load condition (three digits) and 88% of the false control items in the high WM load condition (six digits). This difference was not significant [paired-t_(39)_ =0.443; *p* = 0.660].

Participants of the No WM load group answered 92% of the false control items correctly. This did not differ from the false control item performance of the WM load group [unpaired-t_(36)_ = −1.516; *p* = 0.138].

The results from the false task control items indicate that participants were not simply more accepting of experimental items because of the cognitive burden of the secondary task, but, instead, the difference in acceptance rates between the WM load group and the No WM load group must have a different explanation such as e.g., the costs associated with calculating the interpretation.

#### Response Times of Test Items

**Figure 5:**
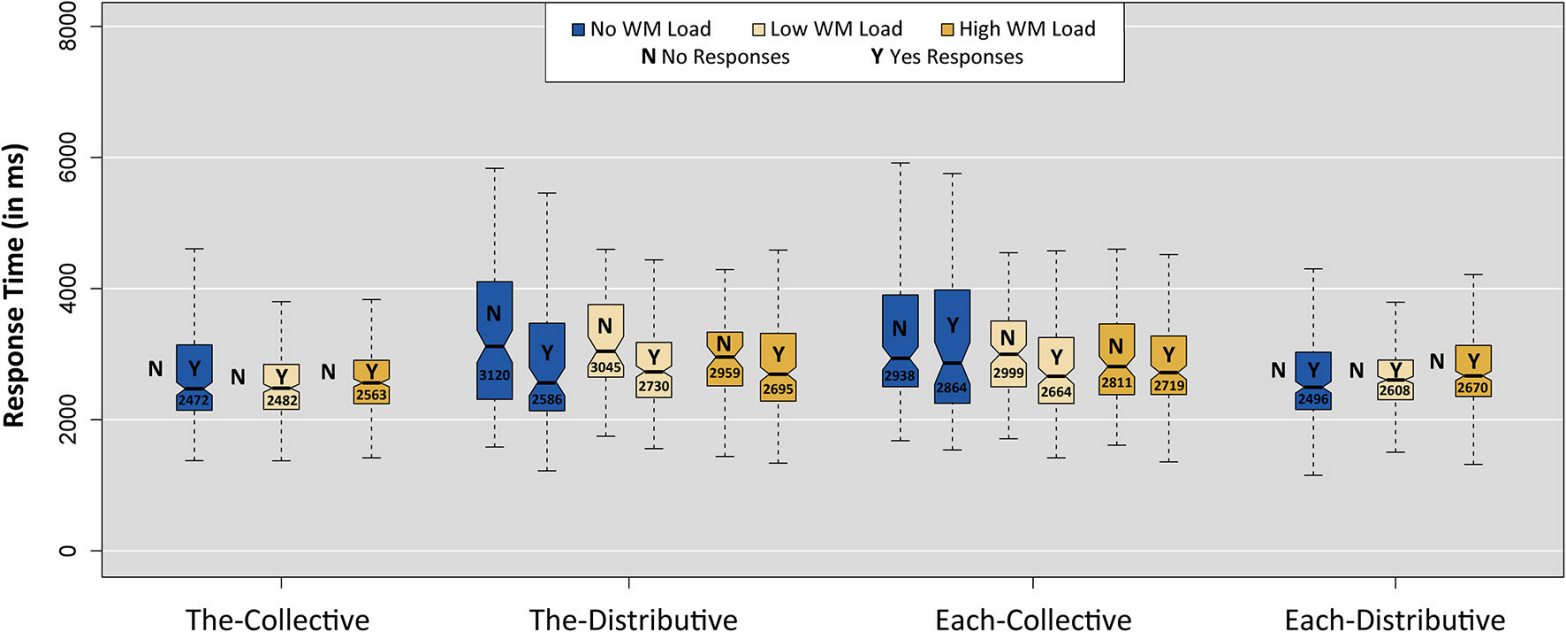
Boxplots of response times per condition and WM load for both “yes” and “no” responses. Notches indicate 95% confidence intervals (Chambers et al., 1983). Note that conditions The-Collective and Each-Distributive had too few “no” responses to be plotted (only seven “no” responses in total).

To test the assumption that implicature calculations require more time (in addition to memory resources), we also analyzed response times (RTs). Figure 5 presents boxplots of RTs for all four linguistic conditions for “yes” and “no” responses separately and per WM load. RTs were measured from the onset of picture and sentence presentation until button press. Outliers were excluded following the interquartile range rule, excluding data points that are more than one and a half times the interquartile range below the first and above the fourth quartile (5.4% of the data was removed).

We performed three different analyses. We first wanted to find out whether it was, indeed, the case that the pragmatic interpretation of the proposed implicature (a “no” response in condition The-Distributive) takes more time than the literal interpretation (a “yes” response in condition The-Distributive). This difference was previously observed by Bott and Noveck (2004) for the “some-not all” implicature.

Second, we wanted to examine whether or not the response times of the pragmatic interpretation were influenced by a WM load. In their dual-task study, De Neys and Schaeken (2007) found that pragmatic interpretations under a high WM load took significantly longer than pragmatic interpretations in the control condition involving only a low WM load.

We also looked more closely into the response pattern of condition Each-Collective. The responses indicated that loading adults' WM not only increased their acceptance of The-Distributive items but also their acceptance of Each-Collective items (see Figure 3). This is unexpected, since we do not predict an implicature in this condition. The verification of this condition is expected to be based on the semantics of Dutch *elke* and should, therefore, not be influenced by a limited WM capacity. Analyzing the response times of condition Each-Collective can show if a similar process underlies the interpretations of the items in conditions The-Distributive and Each-Collective.

We did not analyze the response times of the implicature control items because there were too few items for a proper analysis.

##### Pragmatic vs. Literal Interpretations

To find out whether or not the proposed pragmatic interpretations, indeed, took more time than the proposed literal interpretations, we analyzed the log-transformed RTs of condition The-Distributive, using linear mixed effect modeling [function lmer(): lme 4 package in R, version 3.6.3]. We included the factor response, separating the RTs of the “no' responses from the “yes” responses, since a “no” response indicates a pragmatic interpretation and a “yes” response indicates a literal interpretation.

Based on model comparisons, using the Akaike Information Criterion, the final model (Table 3) included the fixed factors response and block. We also included random intercepts for participants, items, and by-participant random slopes for response and block. The dependent variable was the response time in milliseconds (log-transformed).

**Table 3:** Overview of the model comparing the response times of the pragmatic and literal interpretations, with reference levels: Response: “No,” and Block: 1.

**Predictors**	**Estimate (β)**	**SE**	**df**	***t***	***p***
Intercept	8.127	0.040	47	200.159	<0.001
Response “Yes”	−0.168	0.039	36	−4.288	<0.001
Block 2	−0.165	0.033	92	−4.988	<0.001
Response “Yes” × Block 2	0.124	0.036	99	3.395	<0.001

A main effect of response and block was found, as well as an interaction between the two. Crucially, the main effect of response (β = −0.168; *t*= −4.288; *p* < 0.001) indicates that “no” responses in condition The-Distributive were significantly slower than “yes” responses, following the prediction that pragmatic interpretations take more time than literal interpretations.

The significant effect of the factor block (β = −0.165; *t* = −4.988; *p* < 0.001) indicates that participants were generally faster in block 2 compared to block 1. The significant interaction between response and block (β = 0.124; *t* = 3.395; *p* < 0.001) indicates that the difference between the responses (“yes” and “no”) is smaller in block 2 compared to block 1. These findings could be explained as an effect of task experience. Similar block effects have been found in other dual-task studies (e.g., van Rij et al., 2013).

To be sure that the difference in RTs between the pragmatic and literal interpretation was not caused by the possibility that it would take longer in general to provide a “no” response than a “yes” response, regardless of the tested condition, we also compared RTs on “no” responses in the The-Distributive condition (requiring the hypothesized implicature) to RTs on “no” responses in the Each-Collective condition (not requiring an implicature).

Based on model comparisons, using the Akaike Information Criterion, the final model (Table 4) included the fixed factors condition and block. We also included random intercepts for participants, items, and by-participant random slopes for condition. The dependent variable was the response time in milliseconds (log-transformed).

**Table 4:** Overview of the model comparing the response times of the “no” responses in conditions The-Distributive and Each-Collective, with reference levels: Condition: “The-Distributive,” and Block: 1.

**Predictors**	**Estimate (β)**	**SE**	**df**	***t***	***p***
Intercept	8.159	0.035	49	234.035	<0.001
Condition Each-Collective	−0.061	0.027	28	−2.279	<0.01
Block 2	−0.140	0.018	55	−7.926	<0.001

We found a main effect of condition (β = −0.061; *t* = −2.279; *p* < 0.01), as well as block (β = −0.140; *t* = −7.926; *p* < 0.001). The main effect of block shows that participants' “no” responses were faster in block 2 than in block 1, similar to the previously found block effect. The main effect of condition shows that “no” responses in the Each-Collective condition were significantly faster than “no” responses in the The-Distributive condition. This finding indicates that the difference in RTs between the pragmatic interpretation (“no” response) and the literal interpretation (“yes” response) in condition The-Distributive was not caused by a general difference in RTs between “yes” and “no” responses.

##### Implicature Interpretations Under WM Load

We also examined the influence of a WM load on the response time for rejections of the The-Distributive condition. We, therefore, analyzed the log-transformed RTs of the pragmatic interpretations (“no” responses) of condition The-Distributive, using linear mixed effect modeling [function lmer (): lme 4 package in R, version 3.6.3].

Based on model comparisons, using the Akaike Information Criterion, the final model (Table 5) included the fixed factors wm load and block. We also included random intercepts for participants, items, and by-participant random slopes for block. The dependent variable was the response time in milliseconds (log-transformed).

**Table 5:** Overview of the model examining the influence of WM load on the pragmatic interpretation, with reference levels: WM Load: “No Load,” and Block: 1.

**Predictors**	**Estimate (β)**	**SE**	**df**	***t***	***p***
Intercept	8.219	0.059	32	139.32	<0.001
WM load low	−0.086	0.077	38	−1.120	0.270
WM load high	−0.099	0.077	38	−1.283	0.207
Block 2	−0.155	0.031	224	−4.946	<0.001

The factor wm load was not a significant predictor, showing that there was no difference in RTs between the no WM load condition and both the low WM load condition (β = −0.086; *t* = −1.120; *p* = 0.270) and the high WM load condition (β = −0.099; *t* = −1.283; *p* = 0.207). A releveled model with the low WM load condition as the reference level showed that there was no difference between the low and high WM load conditions either (β = −0.01; *t* = −0.273; *p* = 0.708). These findings are different from the findings by De Neys and Schaeken (2007), who found that pragmatic interpretations under a high load took longer than pragmatic interpretations in the low-load condition. Although not significant, in our model, the estimates of the factor wm load were negative, suggesting that participants became faster under a WM load. This is the opposite direction as the results found by De Neys and Schaeken (2007) where the participants became slower. One reason may be the differences between the tasks. De Neys and Schaeken (2007) used a dot-pattern task, which might have been easier than our digit-span recall task. In our task, the participants may attempt to decrease the WM load by speeding up their responses, thus reducing the period of time during which they need to remember the digits.

The final model did include a main effect of the factor block (β = −0.155; *t* = −4.946; *p* < 0.001), again showing that the participants' response times were lower in block 2, probably as a result of task experience.

##### Response Times in Condition Each-Collective

To check for a difference in RTs within condition Each-Collective, we analyzed the log-transformed RTs of condition Each-Collective, using linear mixed effect modeling [function lmer (): lme 4 package in R, version 3.6.3]. We included the factor response, separating the RTs of the “no” responses from the “yes” responses to be able to find out whether there is a difference in RTs between the different response types, like we found in condition The-Distributive.

Based on step-wise model comparisons, using the Akaike Information Criterion, the final model (Table 6) included the fixed factors response, wm load, and block. We also included random intercepts for the participants, items, and by-participant random slopes for response and block. The dependent variable was the response time in milliseconds (log-transformed).

**Table 6:** Overview of the model comparing the response times of the “yes” and “no” responses in condition Each-Collective, with reference levels: Response: “No,” WM Load: “No Load,” and Block: 1.

**Predictors**	**Estimate (β)**	**SE**	**df**	***t***	***p***
Intercept	8.148	0.047	41	171.948	<0.001
Response “Yes”	−0.044	0.058	34	−0.747	0.460
WM load low	−0.103	0.066	38	−1.558	0.012
WM load high	−0.086	0.071	38	−1.212	0.233
Block 2	−0.185	0.043	65	−4.313	<0.001
Response “Yes” × WM Load low	−0.090	0.081	37	−1.124	0.269
Response “Yes” × WM Load high	−0.057	0.085	40	−0.672	0.505
Response “Yes” × Block 2	0.258	0.076	151	3.380	<0.001
WM Load low × Block 2	0.090	0.084	74	1.070	0.288
WM Load high × Block 2	0.033	0.085	79	0.387	0.700
Response “Yes” × WM load low × Block 2	−0.118	0.117	99	−1.009	0.316
Response “Yes” × WM load high × Block 2	−0.147	0.118	102	−1.247	0.215

Although the final model based on model comparisons included a three-way interaction between the factors response, wm load, and block, we only found a main effect of block (β = −0.185; *t* = −4.313; *p* < 0.001) and an interaction between response and block (β =0.258; *t* = 3.380; *p* < 0.001). Crucially, the factor response did not turn out to be significant, showing that there was no difference in RTs between “yes” and “no” responses in condition Each-Collective. The participants reacted similarly to “no” responses as to “yes” responses. This is in contrast with response latencies in the The-Distributive condition, where we did find a difference between the response types. The difference in response times between conditions The-Distributive and Each-Collective points to a different process underlying the interpretations of the items in both conditions.

## Discussion

In the present study, we investigated whether the adult preference for collective interpretations for distributively unmarked sentences with plural NPs requires working memory resources. We found an effect of loading WM on interpretation preferences.

The-Distributive items were accepted 41% of the time in the No WM load group, a rate comparable to previous adult findings [de Koster et al. (2017) for Dutch; Pagliarini et al. (2012) for Italian]. Crucially, when WM was loaded, participants accepted items of condition The-Distributive at a significantly higher rate (80% for the low WM load condition and 78% for the high WM load condition). These general results are as predicted by the implicature account of distributivity preferences (Dotlačil, 2010). Loading adults' WM elicits a higher rate of acceptance for the The-Distributive condition, that is, the condition argued to involve an implicature.

In the rest of the discussion, we focus on three main issues in interpreting our results. First, we did not find a difference between high and low WM loads, only between the no load and load conditions. Does this matter? And what might explain this result? Second, we unexpectedly found an increase in acceptance for the Each*-*Collective items in the WM load conditions. What could explain this result? And is there evidence that distinguishes it from the increase in acceptance for the The-Distributive items? Finally, a major advantage of the implicature account compared to other explanations for collective and distributive preferences is that it offers an explanation for children's non-adult preferences, since children are known to be less likely to calculate implicatures. But can our results plausibly explain children's very late acquisition of adult preferences?

### Different Load Conditions Effects

Contrary to our expectations, we did not find a difference between the low WM load condition and the high WM load condition. We only found a difference between the two WM load conditions on the one hand the no WM load condition on the other hand. Adults showed greater acceptance of distributive readings without distributive marking in both WM load conditions compared to the no WM load condition.

Similar results have actually been found in another study. van Tiel et al. (2019), who treated no WM load, low WM load, and high WM load, all as between subject conditions, also found a difference between the no load condition and the load conditions for *some-not all*. Note, however, that the other dual task studies that tested only the *some-not all* implicature (De Neys and Schaeken, 2007; Dieussaert et al., 2011; Marty and Chemla, 2013, Marty et al., 2013) did not include a no WM load condition, so we cannot be sure whether or not they might have found a difference between a no load and the low load condition.

There is also some evidence that different implicatures show different sensitivities to cognitive load. We know from previous studies that, even without a cognitive load, implicatures vary in rates of calculation (see, e.g., van Tiel et al., 2016; Sun, 2018). In our study, the low WM load condition was already sufficient to lead to more acceptance of The-Distributive items, and this may be because the proposed scale maybe less common or less automatized than the <some, all> scale. In fact, van Tiel et al. (2019) also found that different implicature types showed varying degrees of sensitivity (or lack of) to WM load. It could be that the less frequent or familiar a scale is, the more sensitive that a scale will be to working memory limitations.

Another factor that may have influenced the effect found was our choice of secondary task. Most other dual-task studies cited [except for Marty et al. (2013) that used backwards letter sequence retrieval, and Ryzhova and Demberg (2020) that used a dot-tracking task] used the dot memory task, where participants had to either recall a very simple dot-matrix pattern of three dots in a vertical or horizontal row, or a more complex pattern of four dots. While the four-dot matrix pattern has been shown to tap executive working memory (see e.g., Miyake et al., 2001) it is not clear to what degree the simpler three-dot matrix pattern actually requires WM resources. In comparison with this three-dot matrix pattern task, our three-digit memory task may load working memory more than the three-dot matrix patterns. Further investigations are needed to know to what degree these different secondary tasks load working memory.

There are, however, two issues we should discuss related to our No WM load group. The number of participants in our no-load group was relatively small (namely, 16, compared with 58 in the load group). While the response rates to the four different conditions tested in the No WM load group are similar to what we have found in previous experiments with adults, using the same visual materials (e.g., de Koster et al., 2018), it is well-known from the implicature literature that the rate of implicature can vary widely, that individuals also may differ in their tendency to calculate implicatures (see, e.g., Feeney and Bonnefon, 2013), and that this can be affected by, e.g., individual differences in working memory capacity (Dieussaert et al., 2011). Despite the large effect size, we have to be cautious about interpreting this comparison, because it could be that our No WM load group was made up of individuals who were particularly predisposed to calculate the implicature, which, in turn, could have made the comparison with the load group particularly significant.

The second issue is that, while the no-load group and load groups were between subjects, the two load groups were within subjects. A stronger comparison could be made if all conditions were run within the participants. However, there are also two problems with doing so. First, the experiment with two conditions was already quite long: Running our two load conditions within the participants already took ~1 h, so running all three conditions as a between-participants study might introduce practice and fatigue effects. Second, exposure to so many items might also lead to unwanted influences and make comparisons between reaction times less valid. This is one of the reasons why van Tiel et al. (2019) ran all three load conditions between subjects. Future work should carefully consider these design issues.

### Each-Collective Items More Acceptable Under WM Load

An unexpected result of our experiment was that, under WM load, adults increased their acceptance of Each-Collective items as well. The effect size is smaller than the increase in the acceptance rate of The-Distributive items (which doubled from 40% to around 80%), but it was still substantial and significant (from 32% to 57%).

In fact, given the literature on quantifiers, which suggests that distributive marking is semantically incompatible with most collective situations, the acceptance rate of 32% for Each-Collective items is actually unexpected. However, our experiments were run in Dutch, and Dutch *elke* has been shown to be closer in interpretation to English *every* than to English *each*. Several experimental studies on Dutch have shown that, while participants strongly prefer distributive meanings with Dutch *elke* in a preference task, they will accept a collective interpretation with *elke* in a picture verification task at relatively high rates (around 35%), contrasting sharply with results with English *each* (Rouweler and Hollebrandse, 2015; de Koster et al., 2017). If Dutch *elke* is better understood as being, in some cases, compatible with both distributive and collective interpretations but with a bias to a distributive interpretation, then one explanation for the effect of WM load may be that the ambiguity resolution process is affected by limited WM capacity, leading some participants to simply abandon disambiguation and simply accept all presented situations with *elke*. Thus, this finding could be similar to the finding of van Rij et al., 2013 that the resolution of ambiguous pronouns is affected by limited WM capacity due to the listener's decreased ability to integrate contextual information needed for the disambiguation.

Note, however, that this explanation is not simply a proposal that a WM load leads to greater acceptance across the board. Instead, the idea is that ambiguity resolution, specifically, may be more sensitive to WM capacity. Recall that participants were not more likely to accept interpretations in general under a WM load, and, for the false task control items, there was no difference in acceptance between the No WM load group and the WM load groups.

Additionally, the RTs for conditions The-Distributive and Each-Collective point to different underlying interpretation processes. Recall that we found out that “no” (pragmatic) responses for The-Distributive items were significantly slower than “yes” (literal) responses. Similar findings have been found in several studies of *some-not all* implicatures, including Bott and Noveck (2004), who found that rejecting upper-bound readings with *some* took longer than the literal, semantic interpretation. If the rejection of distributive readings with unmarked sentences is due to an implicature, it would be consistent with these other results, suggesting implicature calculation also takes longer. However, we did not find any difference in RTs between “yes” and “no” responses for the Each-Collective condition, suggesting that the increased acceptance rate under a WM load is due to a different underlying process more than the increased acceptance found with The-Distributive.

### Children's Non-adult Preferences and the Role of WM

The implicature account of distributive preferences argues that children fail to interpret distributively unmarked sentences as collective because they fail to calculate the implicature. Young children's lower working memory capacity is often used to explain their failure to compute implicatures, so does finding a role for working memory in distributivity preferences offer an explanation for children's late acquisition?

Studies of implicature acquisition for different scales have often found gaps of several years between when children have the lexical knowledge required for an implicature and when they actually compute the implicature. Even for the well-studied *some-not all* implicature, acquisition results seem to suggest that children at age 4 already possess the lexical knowledge necessary for implicature calculation, but many studies find that they do not calculate implicatures consistently until around age seven (e.g., Noveck, 2001; Pouscoulous et al., 2007; Foppolo et al., 2012). Furthermore, previous findings indicate that different implicatures are acquired at different ages. Noveck (2001), for example, examined implicatures based on the <might, must> scale in which the modal *might* implies that the stronger *must* does not hold. He found out that 7-year-olds were the youngest to demonstrate modal competence overall, but that 7- and 9-year-olds still interpreted *might* logically (not implicating “not must”) more often than adults did. This shows that 9-year-old children did not fully master the *might-not must* implicature, yet, though presumably, they already had the lexical knowledge and working memory capacity at age 9 to calculate the *some-not all* implicature. For distributivity, children are non-adult-like in their distributivity preferences until comparatively late. Recall that Dutch children at age 11 were still not adult-like (de Koster et al., 2018) and Italian children at age 14 were also not adult-like (Pagliarini et al., 2012). However, experimental research has shown that, by age nine, children know that lexically distributive markers signal distributivity. At this age, we would expect that children have sufficient working memory capacity for the implicature calculations needed. With such a large gap between acquisition of the lexical scale and adult-like performance in the calculation of the proposed implicature, the role of working memory in the acquisition process is unclear.

One possibility is that working memory capacity is not really the bottleneck in children's late development of collective interpretation preferences. The difficulty is not in the decision to calculate the implicature, or the comparison of alternatives (which may require memory resources) but in recognizing collective and distributive interpretations as comparable alternatives on an informativity scale. Children may need much more verbal experience than what they have at age 9 (or age 11–14) and need to encounter many more examples where the distinction is relevant before they will begin to interpret the two meanings and their potential marking as alternatives on a scale. While many expressions satisfy the requirements to create a scale, only in a context in which the contrast is relevant do implicatures arise. Thus, <car, Honda civic> is a scale, but if a speaker said that a car almost hit them on their morning bike ride, this is unlikely to give rise to the implicature that the car was not a Honda civic, because, in that context, the specific make of the car is irrelevant [see Matsumoto (1995) and Geurts (2010) for more discussion of the contextual constraints on implicatures]. But this also means that, in addition to the recognition of the scale, experience with a weaker term being used in contexts where the contrast with the stronger is relevant is also important, and, for some scales, this might not be all that frequent. Even *some-not all* implicatures, which many researchers believe to be so frequent as to be (almost) a default interpretation, have been found in corpus studies to be much less frequent than previous believed [e.g., see Degen (2015) and Eiteljoerge et al. (2018), who found that only about 15% of uses of *some* in child-directed speech were intended with an implicature meaning]. An additional difficulty could be that, unlike many other scales, the expressions *the* and *each* require different inflectional morphology (e.g., *each* requires a singular verb, and plural definite descriptions require plural verbal morphology in English and in Dutch) and cannot simply be substituted for each other. Even though it is known that substitutability is not a requirement for scalar expressions [e.g., because it does not work in many contexts, e.g., downward entailing environments, see Geurts (2010) for a discussion], it still may influence how easy it is to acquire the scale and associated implicatures. If frequency and experience, indeed, explain children's late acquisition of adult-like preferences, then the lower rate of implicature in adults in our study and children in other studies has different origins: Adults under a working memory load do not calculate the implicature because it requires too many resources. Children do not calculate the implicature because they do not have sufficient experience with the competing alternatives until quite late. The gradual acquisition that we see in children from age nine onwards could then be reflective of a gradual increase in an experience that translates into greater awareness of the scale and thus a greater tendency to recognize the contrast and calculate the implicature.

The alternative explanation is that working memory capacity still does play a role in children's development, and that greater WM capacities, in combination with greater experience, only comes together quite late (e.g., 14+). The advantage of this proposal is that it offers an explanation for the correlation found between working memory and the rate of implicature calculation in de Koster et al. (2017), a relationship that would otherwise be hard to explain if working memory does not play a role at all in children's interpretation processes. More research can, perhaps, help distinguish between these two possible explanations.

## Conclusions and Directions for Future Research

Summarizing, we found that loading adults' WM leads them to accept distributive interpretations without distributive marking, a result that is predicted by the theoretical proposal that adult collective preferences for distributively unmarked sentences originate from a pragmatic implicature. This study thus makes a novel contribution to our understanding of the semantic and pragmatic processes underlying distributivity and their interaction with cognitive resources such as working memory.

Many open questions remain. First, in general, we need more studies looking at other proposed quantity implicatures. Most research has focused on the <some, all> scale and, to a certain degree, the <or, and> scale, but, within the research that has examined the processing of other proposed scales, it does seem that implicatures differ widely in their tendency to be calculated, and their tendency to be sensitive to processing limitations. But we need to confirm this variation experimentally. Second, for distributivity preferences, in particular, it still remains unclear what role working memory capacity plays in children's non-adult interpretation preferences. While working memory capacity was shown to correlate with the rejection of distributive readings without distributive marking in children (de Koster et al., 2018), the very late age at which children begin to be adult-like in their interpretation preferences suggests that other factors, such as experience with the scale or with distributive and collective situations, might play an even bigger role. Investigating this further would help clarify children's development. Another issue is the question of where working memory resources actually come into play in interpretation. Studies such as Marty and Chemla (2013) have found some evidence suggesting that the decision to calculate an implicature may be what requires cognitive resources, but more studies are needed. Future research should develop experiments to try to pinpoint where in the interpretation process resources are required. With more experimental investigations, we can hopefully develop a fuller picture of distributive interpretation preferences.

## Data Availability Statement

The raw data supporting the conclusions of this article will be made available by the authors, without undue reservation.

## Ethics Statement

The study in this article, which involves human participants, was reviewed and approved by the Research Ethics Committee (CETO) of the University of Groningen. The participants provided their written informed consent to participate in this study.

## Author Contributions

AK, JS, and PH conceived the experiment, analyzed the results, read, commented, and revised the manuscript. AK carried out the experiment and wrote the first draft of the manuscript. All the authors approved the final version of the manuscript.

## Conflict of Interest

The authors declare that the research was conducted in the absence of any commercial or financial relationships that could be construed as a potential conflict of interest.

## Footnotes

^1^Because *only some* is semantically equivalent with the some-not all implicature it offers a useful control condition to determine whether the implicature itself requires working memory resources rather than, e.g., verification. Unfortunately, for definite descriptions there is not an obvious semantic equivalent that could be used as a control expression. Future research will have to investigate this question with other methods.

^2^Note however that it is currently a matter of debate whether or not numerals should be expected to give rise to implicatures. See Marty and Chemla (2013) for a discussion.

^3^We thank an anonymous reviewer for stressing this point.
