# Supplementary material for: Child-Like Adults: Dual-Task Effects on Collective vs. Distributive Sentence Interpretations
Source: Front Psychol. 2021 Jun 10;12:556120. doi: 10.3389/fpsyg.2021.556120 (PMC8225266; doi:10.3389/fpsyg.2021.556120)
Supplement: Supplementary file 1 [file Table_1.pdf]

Supplementary Material

1 Supplementary Figures

1.1 Example Pictures used as Implicature Control Items

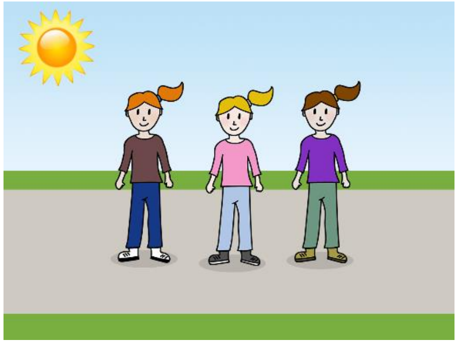

Figure 1 – None of the actors are performing the action.

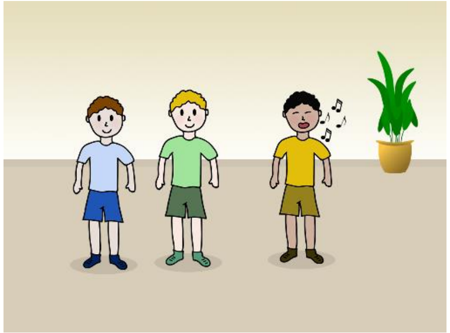

Figure 2 – One of the boys is singing.

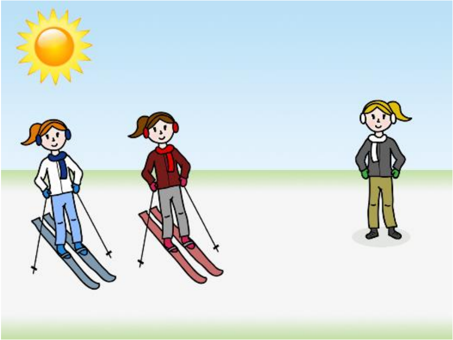

Figure 3 – Two of the girls are skiing.

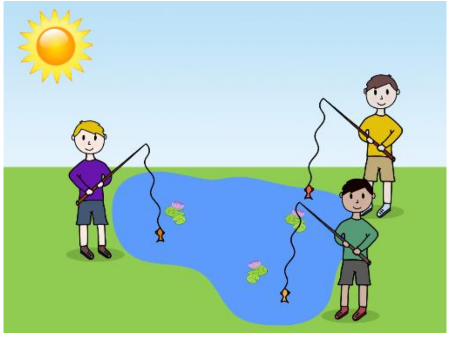

Figure 4 – All three of the boys are fishing.

1.2 Example Pictures used as Task Control Items

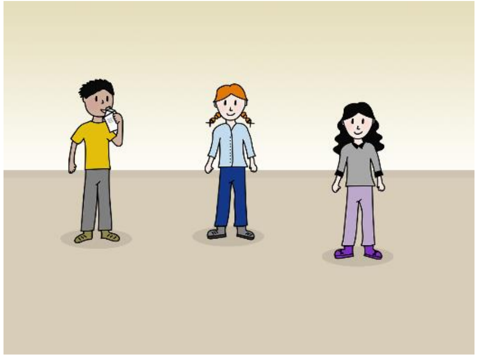

Figure 5 - Picture used with the true Task Control Item for example (8) in the paper (Eng. *The boy is drinking a carton of milk.*)

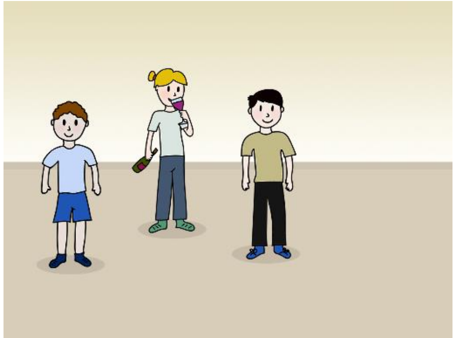

Figure 6 - Picture used with the false Task Control Item for example (9) in the paper (Eng. *The girl is drinking a glass of lemonade*).
